# The Hahnemann Club of Toronto

**Published:** 1888-06

**Authors:** J. D. Tyrrell

**Affiliations:** Secretary


### THE HAHNEMANN CLUB OF TORONTO.

Regular meeting of the Hahnemann Club was held Wednesday, May 2d. Minutes read and adopted. The following papers were read on post-partum hemorrhages. * * * * * Though profitable, our meeting was saddened by the knowledge it was the last one over which our noble and beloved President, Dr. Hall, would preside. The Doctor presented the Club with a large and handsome picture of himself as a souvenir. We were too much surprised to make a suitable acknowledgment on the spot, so a special meeting was called for May 8th, when we surprised our President and teacher by a group picture of *entire* Club, an illuminated address (as inclosed), and a farewell supper.

The subject for next regular meeting will be Convulsions, as follows; Hyosc., Dr. Evans; Opium, Dr. Tyrrell; Helleborus, Dr. E. T. Adams; Apis, Dr. Emory; Nux vom., Dr. Eadie.

J. D. TYRRELL, *Secretary.*

*To John Hall, M. D., Sr., President of "The Hahnemann Club of Toronto:"*

DEAR DOCTOR:—The knowledge that you have decided to leave the home which has been yours for so many years, in which you have built up a lasting reputation as a courteous, skillful, and kind physician; in which, after long years of persevering cultivation and practice in behalf of Hahnemannian Homœopathy, you just begin to see the fruits of your labors in that cause—a small part of which is instanced in this our Hahnemann Club—we say this knowledge has filled our hearts with sadness. The true loneliness we will not fully realize until we miss your presence from our midst, miss your encouraging words, and the benefit of your knowledge and research, of which we have each and all been time and again the favored recipients.

It is afterward that we shall be able to fully appreciate our loss, but trying as our loss will personally be, to our beloved Homœopathy it will be infinitely greater. Standing almost in the presence of your departure, we feel to-night as though that loss can never be filled; that none will arise who will so faithfully and unremittingly preach and practice the truth, and it alone. May the memory of what you have ever tried to accomplish and have accomplished unite and nerve us as a society, and as humble students and practitioners of "Similia," to carry on to victory the flag you have raised, and under which you have so long, honorably, and honestly fought.

We thank you most heartily for the handsome picture of yourself, so thoughtfully and kindly presented. Hanging on the walls of our Club-room, it will be a constant reminder of our beloved President, and act as an incentive to us in our future career.

In return, we would beg your acceptance of this group picture of the Hahnemann Club of Toronto. It is but a slight token of the high esteem and love we bear toward you, and may serve to recall to your mind some of the pleasant (and to us profitable) hours we have passed together. Though absent in body, in mind we will often be with you, and may you ever feel that the hearty good wishes of the Hahnemann Club follow and abide with you for your health, happiness, and prosperity.

Signed,

J. D. TYRRELL, *Secretary*,
L. HAMILTON EVANS,
E. T. ADAMS,
W. J. HUNTER EMORY,
A. B. EADIE.

